# Understanding electrical and chemical transmission in the brain

**DOI:** 10.3389/fncel.2024.1398862

**Published:** 2024-06-26

**Authors:** Dasiel O. Borroto-Escuela, Emmanuell Gonzalez-Cristo, Verty Ochoa-Torres, Emilio M. Serra-Rojas, Patrizia Ambrogini, Luis E. Arroyo-García, Kjell Fuxe

**Affiliations:** ^1^Department of Neuroscience, Karolinska Institutet, Stockholm, Sweden; ^2^Receptomics and Brain Disorders Lab, Department of Human Physiology Physical Education and Sport, Faculty of Medicine, University of Malaga, Málaga, Spain; ^3^Faculty of Engineering and Biotechnology, University OTR and the Regional Cooperative for Comprehensive Medical Assistance (CRAMI), Montevideo, Uruguay; ^4^Cardiology Service, Lozano Blesa University Clinical Hospital, Zaragoza, Spain; ^5^Department of Biomolecular Sciences, Università di Urbino Carlo Bo, Urbino, Italy; ^6^Division of Neurogeriatrics, Department of Neurobiology, Care Sciences, and Society, Karolinska Institutet, Stockholm, Sweden

**Keywords:** chemical transmission, electrical transmission, synaptic chemical transmission, volume transmission, glymphatic system, gap junction, electrical fields, acupuncture

## Abstract

The histochemical Falck-Hillarp method for the localization of dopamine (DA), noradrenaline (NA) and serotonin in the central nervous system (CNS) of rodents was introduced in the 1960s. It supported the existence of chemical neurotransmission in the CNS. The monoamine neurons in the lower brain stem formed monosynaptic ascending systems to the telencephalon and diencephalon and monoamine descending systems to the entire spinal cord. The monoamines were early on suggested to operate via synaptic chemical transmission in the CNS. This chemical transmission reduced the impact of electrical transmission. In 1969 and the 1970s indications were obtained that important modes of chemical monoamine communication in the CNS also took place through the extra-synaptic fluid, the extracellular fluid, and long-distance communication in the cerebrospinal fluid involving diffusion and flow of transmitters like DA, NA and serotonin. In 1986, this type of transmission was named volume transmission (VT) by Agnati and Fuxe and their colleagues, also characterized by transmitter varicosity and receptor mismatches. The short and long-distance VT pathways were characterized by volume fraction, tortuosity and clearance. Electrical transmission also exists in the mammalian CNS, but chemical transmission is in dominance. One electrical mode is represented by electrical synapses formed by gap junctions which represent low resistant passages between nerve cells. It allows for a more rapid passage of action potentials between nerve cells compared to chemical transmission. The second mode is based on the ability of synaptic currents to generate electrical fields to modulate chemical transmission. One aim is to understand how chemical transmission can be integrated with electrical transmission and how putative (aquaporin water channel, dopamine D2R and adenosine A2AR) complexes in astrocytes can significancy participate in the clearance of waste products from the glymphatic system. VT may also help accomplish the operation of the acupuncture meridians essential for Chinese medicine in view of the indicated existence of extracellular VT pathways.

## 1 Introduction

The first neuronal maps and circuits in the central nervous system (CNS) were achieved by Camillo Golgi and Santiago Ramon y Cajal (DeFelipe and Jones, 1992) for which they received the Nobel prize in 1906. The structure and thus the anatomy of the CNS had begun to be understood.

In the beginning of the 1960s the histochemical Falck-Hillarp method for the localization of the monoamines dopamine (DA), noradrenaline (NA) and serotonin in the CNS was introduced (Carlsson et al., 1962; Falck and Torp, 1962; Fuxe, 1963; Anden et al., 1965; Dahlstroem and Fuxe, 1965; Fuxe, 1965b,c). It led to the discovery of the DA, NA and serotonin neurons in the lower brain stem and their monosynaptic and widespread projections and monoaminergic innervation of almost all regions of the CNS (Fuxe, 1965a). This contributed to give further evidence for the existence of chemical neurotransmission in the CNS. The results involved the formation of monosynaptic ascending systems to the tel-and diencephalon and descending monosynaptic systems to the entire spinal cord originating almost exclusively from the lower brain stem (Fuxe, 1965b,c).

CNS communication was early on regarded to take place between neurons, via contacts, named synapses as proposed by Cajal and further established by the electrophysiologist Sherrington outlining their operation via the fast electrical synaptic transmission (Sherrington, 1947).This is the classical fast synaptic transmission with a delay of action usually in the order of 0.3–5 ms.There exists also a presynaptic component, with a varicosity rich in synaptic vesicles, separated by the synaptic cleft from the postsynaptic side, containing different types of post-synaptic receptors and proteins. The neurotransmitters involved were mainly glutamate and GABA. The cleft range varies mainly from 35 to 50 nm.

In the 1960s, it was initially believed that the monoamines were neuroetransmitters and could operate via slow synaptic chemical transmission in the CNS based on histochemistry, biochemistry and pharmacology (Dahlstroem and Fuxe, 1964; Anden et al., 1965; Fuxe, 1965a,b,c; Ungerstedt, 1971; Olson and Fuxe, 1972). At the end of the 1960s and in the 1970s indications were obtained that important modes of chemical monoamine communication in the CNS took place through synaptic and extracellular transmission. This extracellular transmission involves diffusion and flow of transmitters like DA, NA and serotonin (Fuxe and Anden, 1966; Ungerstedt et al., 1969; Fuxe and Ungerstedt, 1970; Fuxe, 1979).

In line with these observations, Descarries found in 1975 (Descarries et al., 1975) using ultrastructural techniques that there exist both junctional and non-junctional monoamine varicosities in the serotonin neurons of the brain. The Falck-Hillarp method and immunocytochemistry also supported the existence of a widespread chemical monoamine transmission including also the serotonin neurons in the CNS (Carlsson et al., 1969; Ungerstedt et al., 1969; Fuxe et al., 1970a,b). In 1986, further evidence was obtained for this extracellular communication involving transmitter and receptor mismatches, previously found by Kuhar et al. (1985) and it was given the name volume transmission (VT) by Agnati and Fuxe and their teams (Agnati et al., 1986; Fuxe and Agnati, 1991). Nicholson and Sykova (1998) have described the extracellular space as having a foam like structure in which migration of VT signals like neurotransmitters, modulators, ions and enzymes can occur. The diffusion and flow in the extracellular space, characterized by the volume fraction, tortuosity, and clearance has had a significant impact on understanding the operation of VT (Nicholson and Phillips, 1981; Nicholson and Sykova, 1998; Hoistad et al., 2002).

In the CNS there is as previously mentioned also another type of synaptic transmission called “electrical transmission.” This electrical transmission permits a direct flow of electrical current from one neuron to the other. One mode is via electrical synapses which are made up of gap junctions, which are low resistance pathways between neurons (Furshpan, 1964; Goodenough and Paul, 2009). It makes it possible for action potentials to pass more rapidly from one nerve cell to another one than in the case for chemical transmission. However, besides being present in all mammalian species they are a minority. The second mode of electrical transmission lacks contacts between nerve cells and involves the ability of synaptic currents to produce electrical fields (Frohlich and McCormick, 2010). These electrical fields can then modulate the synaptic chemical transmission through nerve cells operating via ephaptic transmission (interactions of electrical fields with close by neurons).

The aims of this article involve *inter alia* the understanding how diffusion and flow of transmitters and modulators representing volume transmission (VT), a special type of chemical transmission (Fuxe et al., 2013) can become integrated with electrical transmission. Another aim will be to find out the potential role of the astrocytic aquaporin water channel(AQP) – G protein coupled receptor (GPCR) complexes in mediating the astrocytic transport of waste from the interstitial fluid towards the paralymphatic regions representing part of the glial and lymphatic system (glymphatic system) (Mestre et al., 2020). We will also discuss that VT can mediate the diffusion and flow for transport and signaling in the acupuncture meridians in Chinese medicine (Fuxe et al., 2013; Zhang et al., 2015).

## 2 Chemical transmission in CNS has two modes of operation: synaptic and volume transmission

### 2.1 Synaptic transmission of chemical information

The synaptic transmission between neurons was introduced by Cajal, based on his neuron doctrine which was further developed by neurophysiologists, especially Sherrington (1947). The connectivity is mainly serial with a low divergence and high space filling due to dedicated transmission lines (axons) from one neuron to another neuron. It gives privacy and high safety. The biological effect is phasic. The demonstration of the DA, NA and serotonin (5-HT) neurons in the mammalian brain together with neurophysiology opened up the possibility that chemical transmission had a significant role in synaptic transmission (Fuxe, 1965a,b,c; Anden et al., 1966; Fuxe and Ungerstedt, 1970; Descarries et al., 1975). The concentration of the chemical neurotransmitter in the synapse is usually high (uM). The receptor affinity for the endogenous neurotransmitter is usually low from high nM to uM. The transmission code involves a rate and temporal code. The transmission delay is low and in the millisecond range.

### 2.2 Volume transmission of chemical information

The velocity is slow (seconds-minutes) in VT while synaptic transmission operates rapidly in the millisecond range (Jansson et al., 1999, 2000). The extracellular space is the substrate for VT, which is modulated by the extracellular matrix. In VT, special extracellular fluid pathways exist for diffusion and flow along myelinated fiber bundles and blood vessels (paravascular pathways) using concentration (diffusion), and temperature and pressure gradients (mass movement of a fluid carrying VT signals) (Jansson et al., 1998, 2001; Fuxe et al., 2010).

### 2.3 Astroglia contribution to volume transmission of chemical information

**Figure 1:**
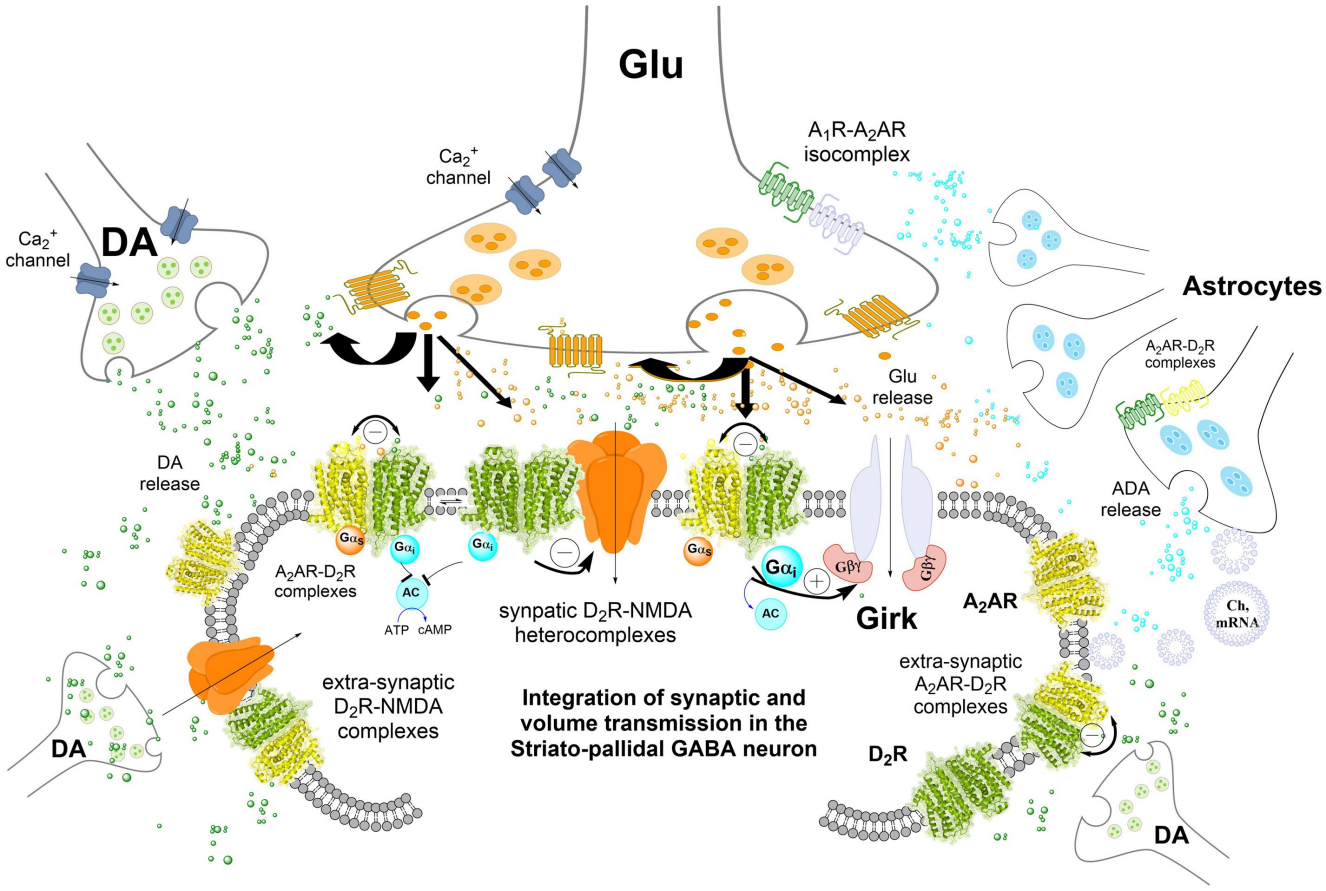
Integration of volume and/or synaptic chemical transmission in the central nervous system (CNS), the striatal-pallidal GABA neuron as an example. Volume and synaptic chemical transmission are essential for CNS function, impacting not only synaptic activity but also the extrasynaptic neuronal membrane. Here, integration can occur also at transcriptional and cellular signaling levels such as phosphorylation. The volume transmission integration extends to interactions between neurons, astrocytes, and other glial cells through this type of chemical transmission, maintaining a balance in nerve cell versus glial cell activity. Astrocytes exert a dominance by modulating blood flow, facilitating mitochondrial, mRNA, receptor and/or chaperone proteins transfer to the neurons, and serving as a primary source of cholesterol. It has become increasingly clear in recent years that integrative receptor-receptor and receptor-protein interactions can exist also in the astrocytes potentially altering the affinity and density of their heterocomplexes. Synaptic transmission, regulated by electrical activity and dependent on calcium influx, involves the release of neurotransmitters triggered by voltage-dependent calcium channels in the presynaptic terminal. These neurotransmitters act on ligand-gated ion channels (e.g., NMDAR) at the postsynaptic membrane, generating a postsynaptic potential. In the striatal-pallidal GABA neurons, dopamine (DA) chemical transmission through synaptic D2Rs also modulates glutamate synaptic transmission. This modulation involves receptor-receptor interactions with synaptic NMDA receptors in heteroreceptor complexes via the NR2B subunit. DA can also diffuse through volume transmission to reach glutamate synapses, where it inhibits NMDAR signaling, thereby reducing synaptic glutamate transmission in these GABA neurons. In addition, extrasynaptic D2 receptors, such as those in A2A–D2 heteroreceptor complexes, further decrease the firing of striatal-pallidal GABA neurons. The inclusion of GIRK channels in the post-synaptic striatal-pallidal GABA neuron was hypothesized based on experimental evidence in striatal medium spiny neurons (MSNs) (Shan et al., 2022).

Other key players in synaptic chemical transmission are the glial cells (astrocytes, oligodendrocites and microglial) (Figure 1). Glial cells serve as a support system for neurons, maintaining their metabolic needs, contributing to ions, neurotransmitters and synaptic homeostasis (Goenaga et al., 2023). In particular, astrocytes play an important role in neuronal activity, synaptogenesis and circuit plasticity (Perez-Catalan et al., 2021; Goenaga et al., 2023). The communication of astrocytes with presynaptic and postsynaptic neurons in the chemical synapses has been very well characterized in recent years and this has brought the concept of “tripartite synapse” (Liu et al., 2021; Perez-Catalan et al., 2021; Goenaga et al., 2023). Moreover, there is evidence that astrocytes also interact with electrical synapses in the optic nerve (Smedowski et al., 2020). Although astrocytes do not respond to electrical stimulation, they respond to fluctuations in calcium levels in a dynamic manner (Perez-Catalan et al., 2021; Goenaga et al., 2023). This astrocytic calcium fluctuation triggers the release of transmitters like glutamate, GABA, D-serine and adenosine triphosphate (ATP), which participate in synaptic communication and plasticity (Liu et al., 2021). Recently, we have shown that astrocytes mediate synaptic plasticity during hippocampal development (Perez-Rodriguez et al., 2019; Falcon-Moya et al., 2020).

### 2.4 Acupuncture meridians

They can represent a form of VT and have the capability to generate VT signals (such as ions, nitric acid, peptides), which could have a major modulation on the surrounding nerve terminals and other types of cells like peripheral blood mononuclear cells modulating the immune system, and mast cells (Fuxe et al., 2013; Zhang et al., 2015). Likely, there is an integration of multiple signals coming from nerves cells, inmmune cells and other type of cells. Short and long-distance acupuncture VT may occur in meridian channels through diffusion and flow in their interstitial fluid. Thus, bidirectional meridian to meriadian communication and integration of signal can be possible through this long-distance VT.

## 3 Volume transmission of chemical information

### 3.1 Observations on monoamine neurons give the first indications of VT

With the demonstration of the central monoamine neurons in the 1960s providing widespread innervation all over the brain and the spinal cord (Carlsson et al., 1964; Dahlstroem and Fuxe, 1964; Fuxe, 1964, 1965b,c; Fuxe and Anden, 1966) and the extracellular release of monoamines induced by amphetamine and putative serotonin reuptake inhibitors (Fuxe and Ungerstedt, 1968, 1970), also a diffuse extracellular mode of monoamine chemical transmission seemed possible. It should be noted that communication via the extracellular fluid pathways appears to be a common mode of transmission in the nervous system of invertebrates (Branton et al., 1978). It can be that synaptic chemical transmission is a later more sophisticated development of special importance for learning and memory (Fuxe et al., 2014a; Borroto-Escuela et al., 2015a, 2016b). It has also been found in ultrastructural work that in monoamine varicosities there are high densities of small vesicles which lack association with synaptic membranes. They can be found in several brain areas (Descarries et al., 1975; Beaudet and Descarries, 1978). Thus, there exist both non-synaptic and synaptic chemical monoamine transmission.

Using the Falck-Hillarp technique, it was possible to early on obtain indication that monoamines can diffuse in the extracellular fluid pathways of the brain (Fuxe and Ungerstedt, 1968; Butcher et al., 1970; Fuxe et al., 1970b). Fuxe obtained indications using the Falck-Hillarp technique (Fuxe and Jonsson, 1973) that DA nerve terminal networks in the median eminence could release DA from varicosities in the median eminence to modulate the release of luteinizing hormone from its varicosities in the median eminence into the portal vessels (Fuxe, 1963; Fuxe et al., 1983). The DA transmitter can also by itself be released into the portal vessels to act as a hormone to inhibit prolactin secretion from the anterior pituitary (Fuxe et al., 2010).

Several proposals were introduced in the 1980s to describe new ways of inter-neuronal communication (Nicholson and Phillips, 1981; Fuxe et al., 2007). The findings of Kuhar (Kuhar et al., 1985) of the existence of transmitter and receptor mismatches are of high relevance. In 1986 we performed a correlation analysis of the regional distribution pattern of central enkephalin and beta-endorphin terminals and of opioid receptors in adult and old male rats (Agnati et al., 1986; Fuxe et al., 1988). We obtained evidence for the existence of two main types of chemical communication in the CNS: the volume transmission and the synaptic transmission (Fuxe et al., 2010). They are in balance with each other and can become integrated at the cytoplasmatic level (Fuxe et al., 2013; Borroto-Escuela et al., 2018a).

It was concluded that in volume VT the transmitter can reach their receptors via diffusion and flow in the extracellular fluid pathways (short or moderate distance) and the cerebrospinal fluid pathways (long-distance). A major impact of VT was that it could mediate not only interactions between nerve cells but also between nerve cell and glial cells and between different types of glial cells. All glial cells can release VT signals, and this is true for all non-neuronal cells in the CNS (Fuxe et al., 2010; Borroto-Escuela et al., 2015a).

### 3.2 Understanding volume transmission

The dopamine, noradrenaline and serotonin neurotransmitters mainly operate via VT. This is also true for all the neuropeptide transitters as well as the ATP and adenosine transmitters. Also the classical synaptic neurotransmitters glutamate, especially upon mGluR activation, and GABA can to some degree signal via VT by entering the extracellular space (Arcos et al., 2003; Borroto-Escuela et al., 2018a). The VT signals involve mainly chemical transmitters and modulators, trophic factors, ions and gases. Energy for signaling is obtained from concentration, temperature, and pressure gradients including also gradients of electrical potentials (charged signals) causing the observed migration (Fuxe et al., 2010). The decoding (GPCR) systems in the targets are mainly the receptors, reuptake proteins and the enzymes. The concentration of the chemical signal at the receptor is often low and in the nm range. The affinity of the receptor for the chemical signal is usually in the nM – pM range. Transmission is dependent *inter alia* on the degree of signal diffusion and the transmission delay is often high from seconds to minutes. There is a reduced space filling in the brain since the extracellular fluid and the cerebrospinal fluid can be used for diffusion and flow of signals with released extracellular vesicles also having a significant role (Fuxe et al., 2013; Borroto-Escuela et al., 2018a). There is a reduced safety in VT versus synaptic chemical transmission in view of frequent long-distance diffusion and flow of chemical signals in the extracellular fluid channels and in the cerebrospinal fluid (Fuxe et al., 2010). There is the VT (Fuxe et al., 2010, 2013) and the classical diffusion parameters like volume fraction, the tortuosity, that shows the increase in path length compared to the strait course and the clearance factor (Nicholson and Sykova, 1998).

### 3.3 Cell types involved in VT and their signaling pathways

In line with these results, it has also been demonstrated that neuroactive compounds can be released from astrocytes to act on neurons in cultures of mammalian brain cells (Nedergaard, 1994; Perez-Rodriguez et al., 2019; Liu et al., 2021; Goenaga et al., 2023). It is clear that also non-neuronal cells can produce VT signals to modulate neuronal signaling. There are also several observations showing that transmitter release from nerve terminals can take place without strict contact with postsynaptic membranes, reaching via VT predominantly extra-synaptic regions. It involves a high frequency of non-synaptic monoamine varicosities (Descarries et al., 2008; Fuxe et al., 2010, 2013).

There is also evidence for a high frequency of non-synaptic varicosities in the cholinergic nerve terminal networks (Umbriaco et al., 1994; Descarries et al., 1998). Thus, VT appears to have a major role also in central cholinergic transmission, especially in cortical cholinergic networks, involving diffusion and flow of acetylcholine. The acetylcholinesterase in the CNS does not seem to be as effective as the one in the peripheral nervous system, allowing an efficient cholinergic VT to develop in the CNS.

### 3.4 Presence of extra-synaptic transmitter receptors and role of astroglia as studied with electron microscopy in VT

Ultrastructural work has repeatedly shown that classical transmitters and peptides to a substantial degree are located in extra synaptic regions, outside synapses, at the presynaptic and postsynaptic levels of neurons (Aoki, 1992; Aoki and Pickel, 1992; Aoki et al., 1994; Sesack et al., 1994). It includes both GPCRs and ionotropic receptors and demonstrates the role of VT in local circuits with short-distance VT. It should be noted that beta adrenergic receptor positive astrocytes exist that can respond to diffusing catecholamines (CA) operating via VT following their release from CA nerve terminals (Aoki, 1992; Aoki et al., 1992; Aoki and Pickel, 1992). The activation of the astrocytic beta-adrenergic receptors can then modulate the astrocytic release of *inter alia* glutamate having an impact on the activity of glutamate synapses of surrounding neurons. Populations of astroglia are important targets for dopaminergic and noradrenergic volume transmission through their contents of dopamine and noradrenaline receptors, including contents of heteroreceptor complexes like A2AR–D2R heterocomplexes (Cervetto et al., 2017, 2023; Pelassa et al., 2019). The monoamine including also serotonin receptors modulate key functions of the astroglia, including microglia (Fuxe et al., 2012; Cervetto et al., 2017; Fuxe and Borroto-Escuela, 2018; Narvaez et al., 2020; Borroto-Escuela et al., 2023).

### 3.5 Volume transmission along extracellular fluid pathways may mediate the diffusion and flow of neurotransmitters and extraneuronal signals in acupuncture meridians

As discussed, volume transmission is a major communication in the CNS involving short and long-distance diffusion and flow of neurotransmitters and glial derived signals in the extra synaptic fluid and the extracellular (interstitial) fluid including also the cerebrospinal fluid for long-distance diffusion and flow (Fuxe et al., 2013). These observations may have relevance for understand the meridian theory of traditional chinese medicine (Zhang et al., 2015). The meridians (interstice) were begun to be understood when VT was introduced and supported by the observations that meridians were found to have low hydraulic resistance channels (Zhang et al., 2015).

### 3.6 VT and the glymphatic system

Glial-lymphatic system is described as representing the astrocytic transport of waste from the interstitial fluid towards the paralymphatic regions (Mestre et al., 2020). This interplay between neurons and astroglia for brain integration and clearance, e.g., in the glymphatic system, is one of the examples that illustrates the key role of VT communication within the CNS. It likely involves allosteric receptor-receptor and receptor-protein interactions (Fuxe et al., 2014a,b; Borroto-Escuela et al., 2015a, 2021; Borroto-Escuela and Fuxe, 2019) to optimize the clearance of metabolic waste from the glial-neuronal circuits (Mestre et al., 2020).

Our hypothesis is that the glymphatic system is part of the extracellular pathways mediating volume transmission, which operates through energy gradients to produce flow (Fuxe et al., 2010; Borroto-Escuela et al., 2015a). As discussed by Prof. Nedergaard and colleagues, there exist aquaporin-4 water channels (AQP4) in high densities on the vascular astrocytic end-feet which increases clearance of the waste in the astrocytic-neuronal networks (Mestre et al., 2020). It is now hypothesized in the current paper that certain types of GPCR like A2AR, D2R, D4R are located on the membrane of vascular astrocytes can form a heterocomplex with AQP4 water channels and other types of water channels. An AQP4 – GPCR receptor-protein complex may enhance the opening of the AQP4 water channels through an allosteric mechanism. This can also increase the passage of fluid through the astrocytes into the interstitial fluid around the glial and neuronal cells inside the blood–brain barrier. It may further improve the flow and clearance of their metabolic products towards the fluid surrounding the beginning of the para venous/para lymphatic regions.

This positive receptor-protein interaction may be in operation mainly at night since the clearance of metabolites may be enhanced in this time period (Mestre et al., 2020). This hypothesis may lead to the introduction of novel treatment of CNS disorders with deficits in removal of waste products in the interstitial fluid of the astrocytic-neuronal networks. Besides influx of fluid over the blood brain barrier through the astrocytic AQP4 water channels modulated by GPCR, we suggest that there also exist in the neuronal-astrocytic network inside the blood brain barrier, AQP4 – GPCR e. g., A2AR heterocomplexes in the astrocytes. In support of this hypothesis, using two-hybrid methods it was demonstrated a potential interactions between GPCR37-like 1 receptor with both, aquaporin 1(AQP1) and aquaporin 10 (AQP10) as well as AQP10 with GPR152 (Luck et al., 2020). It remains to be determined which is the major aquaporin ion channel for participating in the glymphatic system. Through allosteric receptor-protein interactions also these complexes can increase the passage of interstitial fluid through the increased allosteric opening of AQP4 and other water channels on the membrane of astrocytes inside as well as outside the blood brain barrier.

This mechanism can further enhance the flow of interstitial fluid into the extra-cellular pathways from the neuronalglial networks. It will help in the further passage of the interstitial fluid into the para-venous space and the lymphatic vessels (Mestre et al., 2020). This hypothesis can be tested with various types of GPCR complexes to develop a suitable AQP – GPCR complex followed by a pharmacological analysis to optimally increasing the clearance of waste products from the interstitial fluid around astroglia and nerve cells.

### 3.7 Long-distance volume transmission

There are also indications for the existence of long-distance VT based on the presence of mismatches between transmitters and receptors involving the monoamine and peptide systems (Herkenham, 1987; Fuxe et al., 1988; Zoli et al., 1989). Similar binding characteristics in match and mis-match receptors support the view that the mismatch receptors are reached by the diffusing transmitter and can mediate long-distance VT (Jansson et al., 1998, 1999, 2001).

A marked mismatch for DA transmission has also been observed in the retina (Bjelke et al., 1996). The DA nerve cells are in the inner plexiform layer with their DA nerve terminal networks innervating the same layer. Instead, high densities of D1R and D2R are found in the outer plexiform layer representing DA mismatch receptors and mediate DA VT, also found to some extent in the ganglion cell layer and photocell layer (Bjelke et al., 1996). The DA retina VT may have a role in modulating light/dark adaptation.

Long-distance VT for opioid signaling is well illustrated by the beta-endorphin and enkephalin immunoreactive nerve terminal distribution in relation to the distribution pattern of its mu and delta opioid receptors (MOR and DOR) (Agnati et al., 1986; Fuxe et al., 1988). There was a lack of correlation of the distribution of the beta-endorphin and the distribution of MOR and DOR. The mismatches involved long-distances, especially in the cerebral cortex and in the striatum. There are indications that beta endorphin microinjected into the striatum can be detected in the cerebrospinal fluid as an intact beta endorphin which can be detected in the membrane of nerve cell bodies of the globus pallidus, colocated with MOR (Hoistad et al., 2005). It is of high interest that beta-endorphin of the brain formed in the arcuate nucleus (Bloom et al., 1978) can migrate via diffusion and flow in the extracellular fluid and the cerebrospinal fluid to modulate to a large degree via long-distance VT, the function of these opioid receptor subtypes all over the brain and the spinal cord reducing pain and increasing reward leading to addiction development. The work of Bjelke et al. (1996) studying diffusion of dextran in the living brain have also indicated the existence of diffusion and flow of dextran along myelinated fibre bundles and vascular fibres bundles. These can represent fluid pathways for chemical signaling based on long-distance VT.

CSF signaling can also be looked upon as representing long-distance VT, especially prior to synaptic development (Fuxe et al., 2010). Segal has proposed several molecules that can be regarded as relevant CSF signals (Segal, 2000). Of special interest are those with global actions like effects on sleep modulation.

### 3.8 Integration of volume and synaptic chemical transmission of chemical information in the CNS

The major targets for VT signals are various types of GPCR located in the plasma membrane (Figure 1). The neurons in the CNS are therefore continuously modulated by VT signals reaching the plasma membrane where integration with synaptic transmission takes place in addition to integration at the nuclear and cytoplasmatic levels. The main mechanism involved for the integration at the membrane level can be receptor-receptor interactions between synaptic GPCRs and fast synaptic ionotropic receptors, like NMDAR and GABA_A_R, in the synaptic membranes. Such GPCR-ionotropic heterocomplexes were first discovered by Fang Liu and her group (Lee et al., 2002; Liu et al., 2006; Nai et al., 2009) involving e. g., D5R-GABA_A_R, D1R-NMDAR, and D2R-NMDAR. Indications for GABA_A_-D2R receptor-receptor interactions were obtained in striatal membranes already in 1997 based on changes induced in D2R binding affinity (Perez de la Mora et al., 1997). The possible existence of direct interactions between nicotinic and DA receptors in the basal ganglia was discussed in 1995 (Li et al., 1995) and 2008 (Belluardo et al., 2008). It includes also interactions of nicotinic receptors with trophic factors that can underly the neuroprotective effects of nicotine (Belluardo et al., 2008) and interactions of nicotinic receptor with and fast synaptic ionotropic receptors, like NMDAR (Li et al., 2012; Zhang et al., 2016; Jiang et al., 2021). Also, a D2R–nicotinicR and D3R–nicotinicR hetermeric complexes were found in the striatal DA nerve terminals (Quarta et al., 2007; Bontempi et al., 2017). The integration of GPCR and Receptor tyrosine kinases (RTK) in heteroreceptor complexes also has a fundamental role in understanding the mechanism for neuronal plasticity, trophism and modulation of GPCR function (Borroto-Escuela et al., 2012, 2013a,b, 2014, 2015b, 2016a, 2017a,b, 2021; Di Liberto et al., 2017; Di Palma et al., 2020; Ambrogini et al., 2023).

## 4 Integration of chemical transmission with electrical transmission

### 4.1 Interaction of electrical and chemical signals

**Figure 2:**
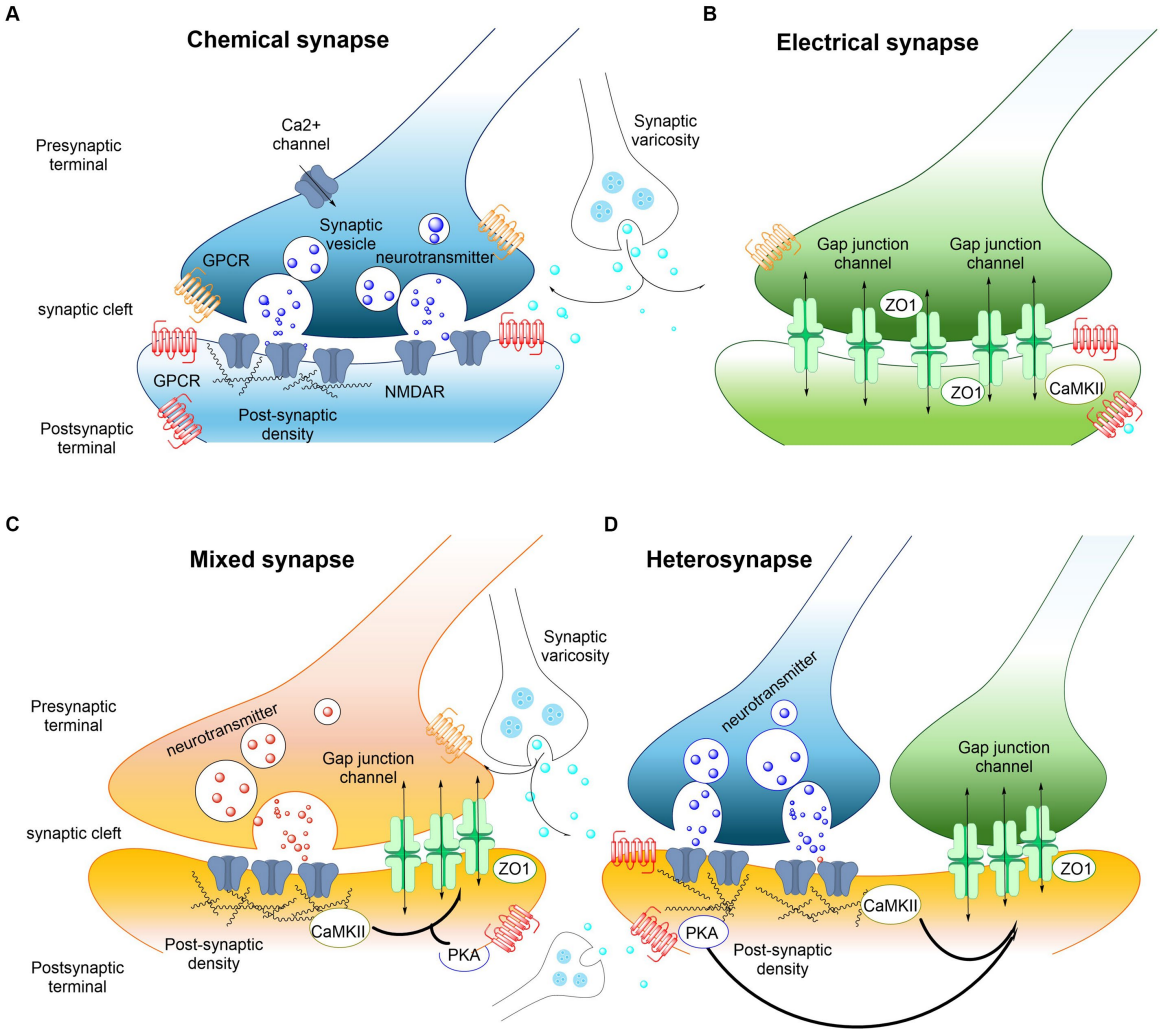
Examples of interactions between electrical and chemical synapses in the CNS. **(A)** Chemical synaptic transmission relies on a complex presynaptic molecular machinery that regulate the probabilistic release of neurotransmitters in a precise and dynamic fashion upon depolarization triggered by an incoming action potential. A similarly complex postsynaptic molecular machinery is essential, including ionotropic and metabotropic receptors. GPCRs (shown in red and orange) are capable of detecting and translating the presynaptic message (neurotransmitters) into various postsynaptic events, ranging from changes in resting potential to alterations in gene expression. The trafficking of glutamate receptors into and out of synapses is regulated, with postsynaptic densities providing scaffolding to manage this process. Key components of these densities include PSD-95 and CaMKII. The regulated trafficking and function of NMDA receptors (NMDARs, shown in blue) are believed to underpin modifications in synaptic strength at glutamatergic synapses. Neurotransmitter modulators released by adjacent synaptic terminals (synaptic varicosities) influence the synaptic strength of both chemical and electrical synapses through the activation of GPCRs, including metabotropic receptors. Regulation at chemical synapses can occur either presynaptically or postsynaptically. **(B)** Electrical transmission is facilitated by clusters of intercellular channels known as gap junctions, which allow direct, bidirectional passage of electrical currents carried by ions (depicted by arrows), as well as intracellular messengers and small metabolites, between adjacent cells. Electrical synapses exhibit bidirectionality: an action potential in the “presynaptic” cell propagates to the “postsynaptic” cell, while the resting membrane potential of the “postsynaptic” cell concurrently influences the “presynaptic” cell (arrows). Proteins within the “semi-dense cytoplasmic matrix” serve as scaffolds, with ZO-1 being a structural component and CaMKII playing a non-essential role in the macromolecular complex of gap junction channels. **(C,D)** Mixed synapses exhibit the coexistence of electrical and chemical synapses. At these synapses, glutamatergic transmission regulates the strength of electrical synapses via a postsynaptic mechanism that involves the activation of NMDA receptors (NMDAR) and CaMKII. Regulation of electrical synapses by glutamatergic transmission could also be formed as a hetero synapse **(D)**. Nearby glutamatergic synapses can regulate the electrical transmission through NMDAR or metabotropic glutamate receptor activation (see also Pereda, 2014).

Electrical synapses are mediated by gap junctions which are aggregates of intercellular channels permitting cell to cell transfer of ions (Figure 2). The gap junctions are built up of connexin proteins (Perkins et al., 1998). Various factors modulate the gap junction channels like voltage, calcium ions and phosphorylation. This modulation makes it possible to open or close the gap junctions (channels), modulating the diffusion and flow of electrical currents between the two cells (Connors, 2017). To date, dendrodendritic, dendrosomatic or somatosomatic electrical signaling via gap junctions is accepted and recognized as an established part in communication of nerve cells in mammals. However, electrical communication can also happen between nerve terminals and postsynaptic elements. The possibility that the presynaptic current can influence the postsynaptic component through gap junctions may occur in specific electrochemical (mixed) synapses (Figure 2). The combination of electrical coupling and neurotransmitter-mediated chemical signaling allows for a complex and dynamic form of communication between neurons where the electrical component of the synapse provides fast and synchronized transmission, while the chemical component offers modulation, plasticity, and the ability to integrate information from multiple sources (Hamzei-Sichani et al., 2012).

Morphologically mixed synapses were first identified by Sotelo and Llinas (1972) in mammals at axon terminals on soma and dendrites. In the classical morphologically mixed synapses, depolarization or hyperpolarization of the postsynaptic nerve cell can have a substantial influence on presynaptic neurotransmitter release through the electrical synapse (Zolnik and Connors, 2016). It is of interest that an axon terminal can form a chemical synapse with one dendrite and can be connected via gap junction also to a second adjacent dendrite located in a parasynaptic position (functionally-mixed synapses) (Sotelo and Korn, 1978). In this way the gap junction may make possible depolarization and calcium influx into the second dendrite, which may cause activation of a silent AMPA receptor in the first dendrite also in a parasynaptic membrane position that may move into a postsynaptic position. It can represent a relevant regulatory mechanism, especially during development with coexistence of electrical and silent glutamate receptors (Peinado et al., 1993; Poncer and Malinow, 2001). In addition, in the first dendrite calcium influx via the postsynaptic NMDAR may modulate the electrical state in the close by gap junction providing one more modulatory mechanism in synapses with mixed morphology (Nagy and Lynn, 2018; Nagy et al., 2019). These observations are of high interest since it opens the possibility that close by dendrites from the same nerve cell can be in dynamic balance with each other through gap junctions, electrically modulating the activity of different glutamate receptors. As a result, homo and heteroreceptor glutamate complexes in these dendrites in parasynaptic positions can become altered in terms of signaling and density through electrical modulation (Figure 2). Thus, electrical, and chemical signal integration can play a significant modulating role in synaptic transmission.

Besides the physical electrical coupling mediated by gap junctions, there exists the ephaptic coupling which involves integration with a close by neuron through the electrical fields produced by their electrical activities (ephaptic transmission). The integration develops when the electrical field of one neuron modulates the electrical potential of another adjacent neuron. It leads to altered excitability with potential synchronization of their activities (Anastassiou et al., 2011). Furthermore, the diffusion of charged neurotransmitters with or without coupling to membrane receptors, can via volume transmission in the extracellular space form electrical fields (Savtchenko et al., 2004). It should be noted that the volume transmission can involve spread of electrical currents between nerve cells via the extracellular fluid through the electrogenic activity of neurons, modulating their activity, membrane potential and receptor function (Jefferys, 1995). Therefore, electrical fields of synaptic currents can become integrated with chemical transmission including both synaptic and volume transmission. The chemical transmission has the ability to integrate information from different types of receptors participating in chemical or electrical transmission. It includes ionotropic receptors and/or GPCRs and/or RTKs through their allosteric receptor-receptor interactions with each other, giving high plasticity to these types of synapses (Fuxe and Agnati, 1985; Borroto-Escuela et al., 2012; Fuxe et al., 2014a). The various types of receptor-receptor interactions can, e.g., lead to local activation or inhibition of transmitter or modulator release, altering diffusion of charged transmitters. Changes in in the presynaptic potential could also be involved at the site of transmitter release. Changes in electrical fields may also be considered in these events through alterations in polarization.

### 4.2 Possible voltage dependence of GPCR

While many ion channels have long been known to be voltage-sensitive, this property has not been attributed to GPCRs until quite recently. A notable example was the discovery in 2003 that M2 muscarinic acetylcholine receptors (M2R) display depolarization-induced decreases in agonist binding and functional potency (Ben-Chaim et al., 2003). M2R voltage sensitivity has been implicated in the autoreceptor function of this GPCR, by permitting rapid control of neurotransmitter release kinetics by membrane voltage. The agonist binding affinity and the activity level of some GPCRs, e.g., metabotropic mGluR3 and mGluR1a (Ohana et al., 2006), and alpha2A adrenergic receptors (Rinne et al., 2013) were shown to be also regulated by membrane potential *in vitro*, suggesting a voltage-dependence of these receptors (Ben-Chaim et al., 2003). Additionally, using Förster resonance energy transfer-based (FRET) biosensors in patch clamp experiments, it was discovered that prostanoid receptors exhibit a robust voltage dependence at the receptor level as well as in downstream signaling (Kurz et al., 2020). Agonist-mediated activation of prostaglandin F receptors and prostaglandin E(2) receptors as well as thromboxane receptors are activated upon depolarization, whereas prostacyclin receptors are not. The discovery that GPCRs are voltage-sensitive has improved our understanding of their behavior. For instance, the M2R was found to exhibit depolarization-induced charge movement associated currents, implying that this prototypical GPCR possesses a voltage sensor (Ben-Chaim et al., 2003). However, the typical domain that serves as a voltage sensor in voltage-gated channels is not present in GPCRs, making the search for the voltage sensor in the latter challenging (Barchad-Avitzur et al., 2016; Rozenfeld et al., 2021).

Although many of the physiological and pharmacological implications of this effect remain unclear, the demonstrated ability of depolarization to potentiate GPCRs at near threshold agonist concentrations represents a novel mechanism for chemical and electrical signal integration (Gurung et al., 2008).

In recent years, a physiological role for the voltage dependency of GPCRs has been identified by demonstrating crucial involvement of GPCR voltage dependence in neuronal plasticity and behavior (Rozenfeld et al., 2021). Studies on muscarinic receptors by Rozenfeld et al. suggested that GPCR voltage dependency plays a role in many diverse neuronal functions, including learning and memory.

Sahlholm et al. (2008a,b,c) further investigated voltage sensitivities of D2-like dopamine receptors (D2R, D3R, D4R) using Xenopus oocytes expressing DA-like receptors with G protein inwardly rectifying potassium (GIRK) channels as readouts. It was found that the dopamine potency was reduced by depolarization to a similar extent in both isoforms of the D2R (Sahlholm et al., 2008a). However, the dopamine D3R potency was not significantly affected, while a weak, albeit significant decrease in potency was observed with the dopamine D4R (Sahlholm et al., 2008a,c). Thus, a differential voltage sensitivity was observed. In mammalian cells, changes in FRET were used as a readout for D2(short)R activation, showing similar potency shifts as observed with GIRK channels (Sahlholm et al., 2008b). Furthermore, radioligand binding experiments carried out on oocytes in hyperpolarizing vs. depolarizing buffer established that dopamine binding is reduced by depolarization. Voltage effects varied among agonists at D2(short)R, independent of G protein subtypes. Specific agonist-receptor interactions, particularly involving hydroxyl and amine groups, influenced voltage-induced potency shifts, highlighting differential voltage sensitivity among D2-like receptors (Sahlholm et al., 2008b). This underscores the relevance of GPCR voltage sensitivity in dopaminergic signaling, and reveals insights into voltage-sensitive agonism, and suggests using differentially voltage-modulated agonists to explore this phenomenon in native tissue and for and for drug development.

More recently, Ambrogini et al. (unpublished findings) evaluated the voltage sensing properties of adenosine A2AR and dopamine D2R in HEK 293 cells, single transfected with A2AR or D2R. The results indicated that both A2AR and the D2R lacked voltage dependence. These results suggest that for at least these two GPCRs in the model and protocol used, there is a lack of integration between chemical and electrical transmission. However, in view that the HEK-293 cells express endogenously both adenosine A2AR and dopamine D2R, there is a possibility that the existence of A2AR–D2R heteroreceptor complexes may have a major role on the control of these promoters voltage sensing properties. It is demonstrated that A2AR and D2R can form heteroreceptor complexes with antagonistic allosteric receptor-receptor interactions. Thus, the possible voltage dependence of D2R, previously demonstrated, would be affected within the A2AR–D2R heteroreceptor complexes. We cannot exclude also that the intrinsic voltage sensing properties observed in GPCRs, may depends on several other factors such as the expression level of G protein subunits and their stoichiometry, and/ or the GPCR-lipid interaction (e.g., cholesterol).

## 5 Concluding remarks

Volume and synaptic chemical transmission plays a highly significant role in the CNS and become integrated not only in the synapses but also in the neuronal cytoplasm through integration at the level of transcription and cellular signaling like phosphorylation. The integration of neurons and astrocytes and other types of glial signaling takes place via volume chemical transmission enabling a balance in the activity of nerve cells versus glial cells. The astrocytes in dominance, can modulate blood flow and mediate transfer of mitochondria to neurons and is the major source of cholesterol (Siracusa et al., 2019). It has become increasingly clear in recent years that integrative receptor-receptor and receptor-protein interactions can exist also in the astrocytes potentially altering the affinity and density of these heterocomplexes (Cervetto et al., 2017; Narvaez et al., 2020; Borroto-Escuela et al., 2023) as previously demonstrated in neurons (Borroto-Escuela et al., 2012, 2014, 2017c, 2018b, 2023; Chruscicka et al., 2019; Di Palma et al., 2020; Chruscicka et al., 2021; Romero-Fernandez et al., 2022).

Electrical transmission becomes integrated with chemical transmission through its electrical synapses formed by gap junctions, which elicit ion channels between two nerve cells through their low resistance. Presynaptic currents can modulate the postsynaptic part via gap junctions in electro-chemical synapses. Another integration mechanism is that electrical fields generated by synaptic currents through ephaptic transmission can modulate electrical currents and rhymes in adjacent nerve cells.

As to the glymphatic system, AQP water channels–GPCR complexes may enhance the opening of e. g. the AQP4 water channels through the allosteric interactions in the complex involving e. g. the A2AR. This increased passage of fluid through the astrocytes outside and potentially inside the blood brain-barrier may improve the flow and clearance of the metabolic waste products by enhancing their passage to the para-venous regions. This hypothesis will be further tested in future experiments, including pharmacological experiments. It underlines the potential role of volume transmission combined with e. g. AQP4–A2AR complexes in the clearance of waste products in the glymphatic system. The potential existence of volume transmission along acupuncture meridians may open a up a new understanding of the mechanism for the operation of the acupuncture meridians in chinese medicine.

## Author contributions

DB-E: Conceptualization, Funding acquisition, Investigation, Resources, Supervision, Visualization, Writing – original draft, Writing – review & editing. EG-C: Writing – original draft, Writing – review & editing, Conceptualization. VO-T: Writing – original draft, Writing – review & editing, Conceptualization. ES-R: Writing – original draft, Writing – review & editing, Conceptualization. PA: Writing – original draft, Writing – review & editing. LA-G: Writing – original draft, Writing – review & editing, Conceptualization. KF: Conceptualization, Funding acquisition, Resources, Supervision, Visualization, Writing – original draft, Writing – review & editing.
